# Revolutionizing Dupilumab Treatment in Refractory Eosinophilic Cellulitis: A Case Report and Comprehensive Literature Review

**DOI:** 10.7759/cureus.50333

**Published:** 2023-12-11

**Authors:** Divya Shah

**Affiliations:** 1 Internal Medicine, University of Arizona College of Medicine - Phoenix, Phoenix, USA

**Keywords:** eosinophilic infiltrates, refractory skin lesions, wells syndrome, eosinophilic cellulitis, dupilumab

## Abstract

Eosinophilic cellulitis (EC) or Wells' syndrome, characterized by its rarity and diverse clinical presentations, presents substantial challenges in diagnosis and treatment. This case study details the challenging journey of a 48-year-old woman with non-specific skin lesions, showcasing the persistence of dermatitis despite exhaustive attempts at various interventions outlined in the literature. In the absence of a consensus on optimal management for EC, this study introduces a transformative solution through the utilization of dupilumab, a monoclonal antibody targeting interleukin-4/interleukin-13. The patient experienced marked improvement and resolution of dermatitis following dupilumab treatment alone, highlighting the efficacy of this targeted immunomodulatory approach in cases refractory to traditional therapies. This pioneering case underscores the significance of exploring innovative treatments and suggests dupilumab as a potential breakthrough therapeutic option in addressing EC refractory to standard medical management.

## Introduction

Eosinophilic cellulitis (EC) also known as Wells’ syndrome is a rare inflammatory skin disorder with variable clinical presentations, which lacks specific diagnostic tests and standardized criteria, thus complicating identification and confirmation. Diagnosis involves clinical evaluation, skin biopsies, and blood tests, given its resemblance to other skin conditions. The rarity of EC and the absence of standardized diagnostic criteria contribute to the complexity of its diagnosis. Additionally, the treatment poses challenges due to the scarcity of evidence-based guidelines, thereby relying on individual experiences and case reports [1,2]. Standard treatments with variable effectiveness include oral corticosteroids, topical steroids, immunosuppressive agents, and antihistamines. The absence of a universally accepted treatment protocol underscores the need for further research. This case study presents successful monoclonal antibody dupilumab use, contributing to the exploration of alternative therapies for eosinophilic cellulitis, particularly in cases resistant to conventional treatments.

Dupilumab, a monoclonal antibody, targets interleukin-4 (IL-4) and interleukin-13 (IL-13), critical proteins in inflammatory processes associated with various allergic and immune-mediated conditions. Dupilumab modulates the immune response by inhibiting these cytokines, effectively reducing inflammation. Being primarily used for moderate to severe atopic dermatitis in adults and adolescents unresponsive to other treatments, dupilumab has also gained approval for moderate to severe asthma and chronic rhinosinusitis with nasal polyposis [1]. Its mechanism of action positions it as a promising therapeutic option for conditions characterized by dysregulated immune responses and inflammation, offering a targeted approach to address the underlying causes of these disorders.

This case was previously presented as a poster at the 2023 American College of Allergy, Asthma, & Immunology Annual Meeting on November 10, 2023, and then published as a brief abstract in the supplement of the November 2023 edition of the *Journal of Allergy and Clinical Immunology*.

## Case presentation

**Figure 1:**
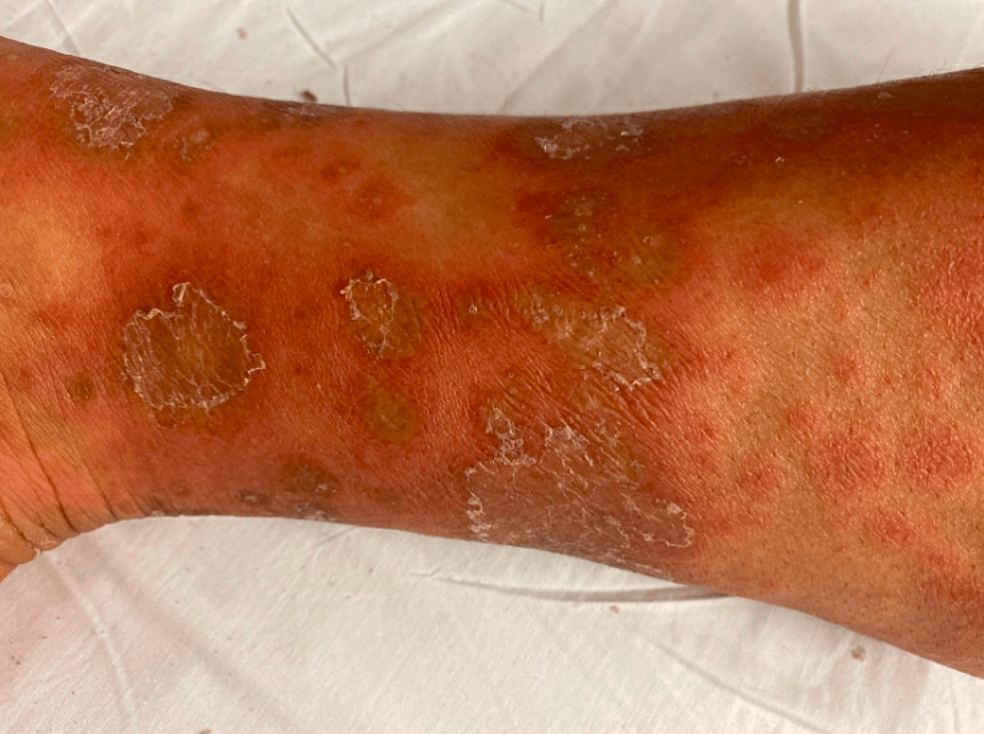
Skin findings on physical exam upon initial presentation

In this case presentation, a 48-year-old woman with a history of allergic rhinitis sought evaluation for a persistent rash that had lasted for four months. Physical examination revealed annular, raised, and erythematous lesions with central hyperpigmentation located on the limbs (Figure 1), while the remainder of the examination was unremarkable. The initial assessment of the patient's symptoms considered potential causes such as bacterial, fungal, or parasitic infections as well as conditions like Churg-Strauss Syndrome and bullous pemphigoid for a comprehensive differential diagnosis. Despite the initial suspicion of a fungal lesion, treatment with griseofulvin showed no improvement. Subsequent courses of doxycycline and minocycline, prescribed for suspected cellulitis, failed to alleviate the rash, which had extended to her bilateral lower extremities and abdomen in a scattered distribution. Topical steroids, tacrolimus, and various antihistamines were then prescribed, yielding no significant improvement. Routine laboratory values were within normal limits, and extensive rheumatologic and infectious workups were unremarkable (Table 1).

**Table 1:** Summary of significant laboratory findings L: Liter; IU: International unit; mL: Milliliter; mm: Millimeter; h: Hour; mg: Milligram; dL: Deciliter.

Lab variables	Lab values	Reference ranges
White blood cells	13 × 10^9 ^cells/L	4.5 to 10.0 × 10^9^cells/L
Eosinophil %	6%	1%-4%
Serum IgE	120 IU/mL	<100 IU/mL
Erythrocyte sedimentation rate	32 mm/h	<20 mm/h
C-reactive protein	12 mg/dL	<0.3 mg/dL

**Figure 2:**
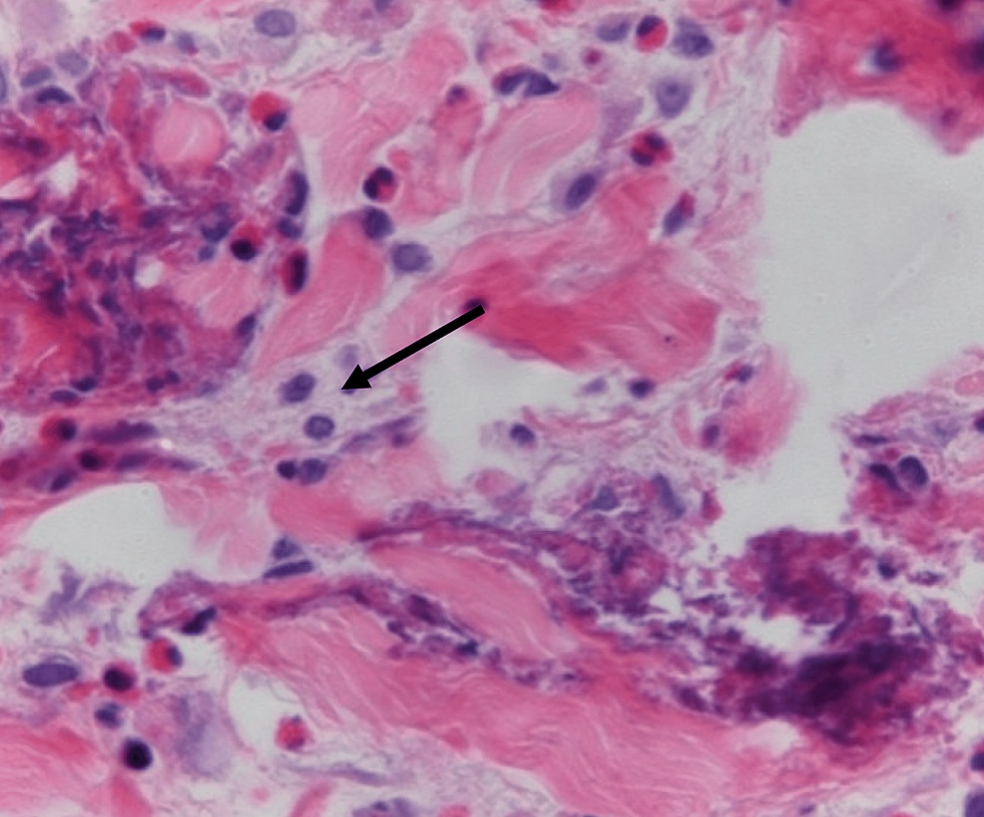
Skin biopsy results exhibiting the presence of dermal infiltration by eosinophils Source: This image was adapted with permission from Traidl et al.

Considering the persistent nature of the condition, a skin biopsy was performed, revealing interstitial infiltration with abundant eosinophils (Figure 2) [3]. The diagnosis of EC was established, prompting the initiation of systemic corticosteroids and cyclosporine. Although the patient experienced mild symptom resolution, the rash recurred. Additional trials of methotrexate and colchicine were attempted without success [1,2]. Considering the potential efficacy suggested by prior studies, monoclonal antibody therapy targeting IL-4/IL-13, specifically dupilumab, was introduced. The rationale behind this choice was based on dupilumab’s mechanism of action in modulating the immune response and reducing inflammation. Remarkably, the patient reported a notable improvement in her dermatitis after a single dose of dupilumab. Despite subsequent attempts to transition to mepolizumab, an anti-IL-5 agent, based on cases indicating its efficiency in EC, the patient experienced several flare-ups during the trial and was ultimately restarted on a regimen of 200 mg dupilumab subcutaneously every two weeks for six months [4]. This unique case underscores the challenges in managing EC and suggests the potential superiority of dupilumab over mepolizumab.

## Discussion

EC poses diagnostic challenges due to its rarity and absence of specific markers, while treatment is complicated by variable responses to standard therapies, necessitating further research and individualized approaches. The lack of large-scale clinical trials, often relying on case reports and small observational studies, reflects the rarity of EC. Commonly, oral and topical corticosteroids are the initial treatment for the inflammatory component of EC, with immunomodulators like methotrexate and cyclosporine showing success in some cases [1]. Antihistamines are used to manage associated itching; even unconventional treatments like colchicine have demonstrated resolution in specific cases [2]. This study highlights a case of EC refractory to standard medical management, emphasizing the rarity and complexity of EC as key challenges. The limited understanding of its pathogenesis and the absence of well-established treatment protocols contribute to inconsistent therapeutic outcomes. The diverse clinical manifestations further complicate treatment choices, and the variable responses to traditional interventions underscore the necessity for individualized strategies. The patient’s lack of response to multiple standard treatments underscores the need for alternative therapeutic approaches and emphasizes the ongoing research required to enhance our understanding of this rare dermatologic condition.

**Figure 3:**
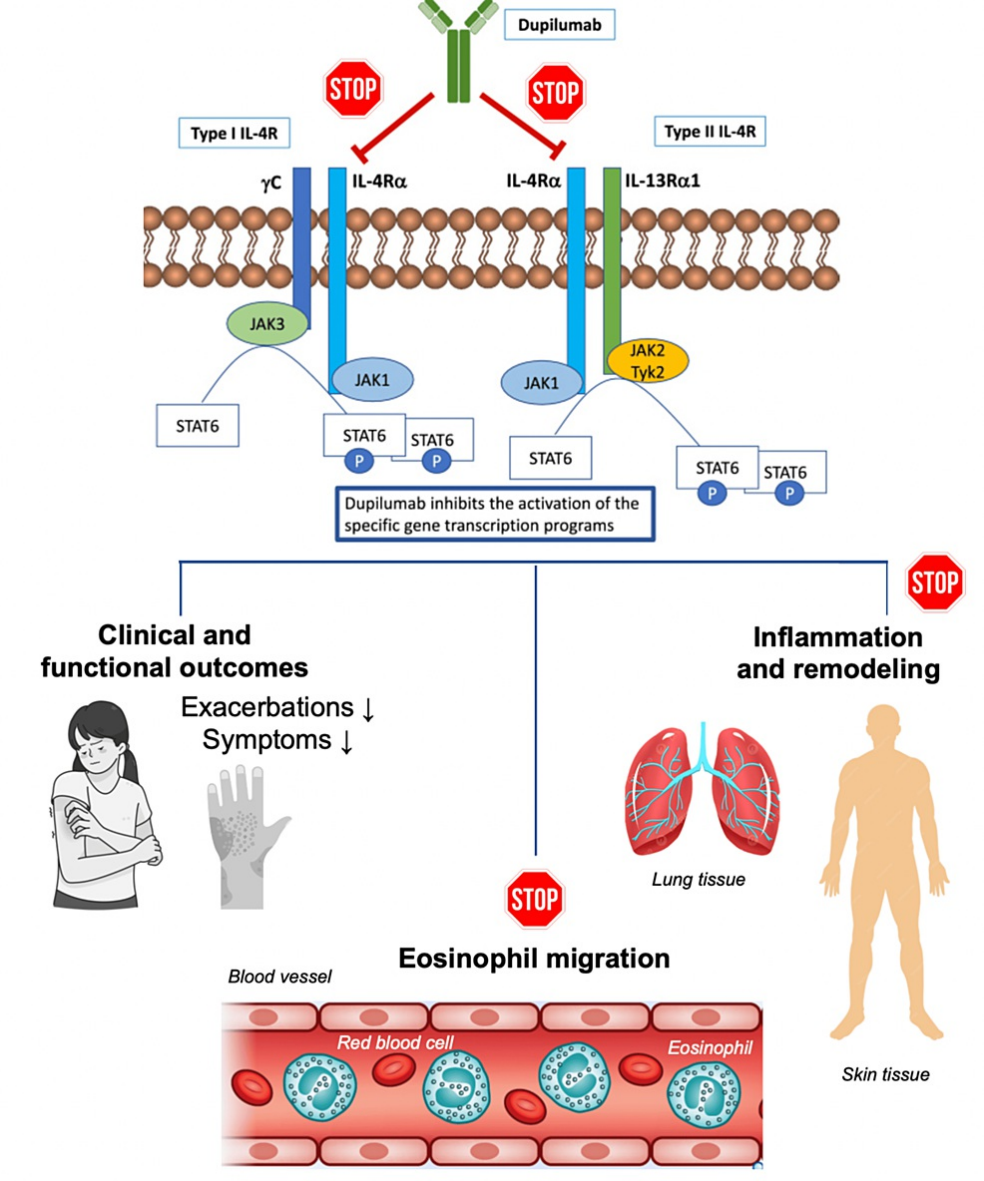
Mechanism of action of dupilumab

The documented improvement of EC with dupilumab is considered a significant breakthrough in managing this challenging dermatologic condition. Dupilumab’s potential effectiveness is attributed to its targeted mechanism of action as a monoclonal antibody, which explicitly inhibits IL-4 and IL-13, crucial cytokines involved in the inflammatory response. In EC, where eosinophils play a central role, dupilumab’s targeted cytokine inhibition is believed to modulate eosinophilic activity, regulating the immune response and reducing eosinophil influx into the skin (Figure 3). The ineffectiveness of mepolizumab, targeting IL-5, suggests the involvement of immune mechanisms beyond IL-5 in EC’s pathogenesis, highlighting the disease’s complexity. The author acknowledges three additional cases of EC successfully treated with dupilumab, supporting the growing evidence of its efficacy in addressing the challenges of EC [5-7]. While these studies collectively underscore the potential efficacy of dupilumab in treating EC, the presented study holds a unique position. The highlighted study focuses specifically on a patient refractory to standard management, providing a clearer evaluation of dupilumab’s impact in challenging cases of EC. This distinction sets it apart from the other cases where dupilumab was introduced as part of the treatment regimen alongside conventional therapies, complicating the assessment of the singular impact of dupilumab versus other treatments. The emphasis on a patient resistant to standard management adds valuable insights into the potential significance of dupilumab as a targeted therapeutic option for cases where traditional treatments have proven insufficient. Overall, this study contributes to understanding dupilumab’s role in managing EC and highlights its potential as a promising treatment avenue in challenging cases of Wells’ syndrome.

## Conclusions

This case marks the first reported successful management of EC with dupilumab, signifying a breakthrough in addressing the formidable challenges posed by this dermatologic disorder. EC is known for its elusive diagnostic criteria and resistance to standard medical treatments, compounded by uncertainties in its pathogenesis and a lack of consensus on optimal therapeutic strategies. The study’s significance lies in shedding light on the potential efficacy of dupilumab, providing hope for patients resistant to conventional therapies. The documented success underscores the importance of exploring targeted and innovative approaches for EC, emphasizing the need for further research and clinical investigation to validate and build upon these promising findings.
